# Fano Resonance in Near-Field Thermal Radiation of Two-Dimensional Van der Waals Heterostructures

**DOI:** 10.3390/nano13081425

**Published:** 2023-04-20

**Authors:** Huihai Wu, Xiaochuan Liu, Keyong Zhu, Yong Huang

**Affiliations:** School of Aeronautic Science and Engineering, Beihang University, Beijing 100191, China

**Keywords:** near-field radiative heat transfer, two-dimensional van der Waals heterostructure, Fano resonances, electromagnetic-induced transparency

## Abstract

Two-dimensional (2D) materials and their vertically stacked heterostructures have attracted much attention due to their novel optical properties and strong light-matter interactions in the infrared. Here, we present a theoretical study of the near-field thermal radiation of 2D vdW heterostructures vertically stacked of graphene and monolayer polar material (2D hBN as an example). An asymmetric Fano line shape is observed in its near-field thermal radiation spectrum, which is attributed to the interference between the narrowband discrete state (the phonon polaritons in 2D hBN) and a broadband continuum state (the plasmons in graphene), as verified by the coupled oscillator model. In addition, we show that 2D van der Waals heterostructures can achieve nearly the same high radiative heat flux as graphene but with markedly different spectral distributions, especially at high chemical potentials. By tuning the chemical potential of graphene, we can actively control the radiative heat flux of 2D van der Waals heterostructures and manipulate the radiative spectrum, such as the transition from Fano resonance to electromagnetic-induced transparency (EIT). Our results reveal the rich physics and demonstrate the potential of 2D vdW heterostructures for applications in nanoscale thermal management and energy conversion.

## 1. Introduction

Radiative heat transfer between two objects in far-field contexts is bound by Stefan Boltzmann’s law of the black body. However, when the separation distance is less than the characteristic wavelength predicted by Wien’s law, the near-field radiative heat transfer is significantly enhanced due to photon tunneling of evanescent waves, exceeding the blackbody limit by several orders of magnitude, especially when the material supports surface wave resonances (such as surface plasmon polaritons and surface phonon polaritons) [1,2,3]. In recent decades, near-field thermal radiation has attracted much attention for its potential applications in areas such as thermal management [4,5,6,7], thermal lithography [8], nanoscale thermal imaging [9,10], thermally assisted magnetic recording [11], and energy conversion (e.g., thermophotovoltaics) [12,13,14,15,16]. However, the active control of near-field thermal radiation is still a key challenge.

Two-dimensional (2D) materials provide an excellent platform for the enhancement and manipulation of near-field thermal radiation due to their strong light-matter interactions in the infrared and have been attracting considerable attention in the last decade, including graphene [17,18,19,20,21,22,23,24,25], black phosphorus [26,27], and transition metal dichalcogenides [28]. Additionally, graphene can be stacked with other two-dimensional (2D) crystals to form two-dimensional van der Waals (2D vdW) heterostructures like LEGO blocks that exhibit novel optical responses [29], which provide rich opportunities for light-matter interactions and show great potential in the near-field thermal radiation. However, the near-field thermal radiation of such 2D vdW heterostructures has not received much attention up to this point [30].

Graphene and 2D hBN, with the same crystal structure and naturally small lattice mismatch, are considered fundamental building blocks for 2D heterostructures. Graphene supports surface plasmon polaritons [18,23,24] and the hybrid polaritons formed by their coupling with phonon polaritons in polar substrates (if present) [20,31,32,33,34], which can mediate and strongly enhance near-field thermal radiation. The radiative spectrum and heat flux can be actively controlled by changing its chemical potential (by chemical doping or by applying a gate voltage in experiments [35,36]). 2D hBN is the monolayer limit of bulk hBN, which support hyperbolic phonon polaritons within two broad Reststrahlen band, resulting in a significant enhancement of the near-field thermal radiation [37,38,39,40]. However, 2D hBN supports 2D phonon polaritons that differ from those in bulk hBN, owing to the degeneracy of the longitudinal optical (LO) and transverse optical (TO) phonon modes at the Γ point, leading to the absence of LO-TO splitting and the disappearance of hyperbolic modes [41,42,43,44].

In this paper, we investigate the near-field thermal radiation of 2D vdW heterostructure vertically stacked by these two single-layer materials, focusing on the radiative spectral properties. We have demonstrated the contribution of the coupling between broadband plasmons in graphene and narrowband phonon polaritons in 2D hBN to the near-field radiative heat transfer and found an asymmetric line shape in the spectra, and confirmed it to be a Fano resonance arising from the interference between the continuum state (plasmons) and the discrete state (phonon polaritons). The Fano resonance has been widely discovered in spectra of various photonic nanostructures [45,46] as well as in the near-field radiative spectra of bulk materials [47,48], offering promising applications in optical switches, sensing, and other fields. We also show that by tuning the chemical potential of graphene, we can modulate the spectra, such as the transition from Fano resonance to electromagnetic-induced transparency (EIT), and can actively control the radiative heat flux. Our findings highlight the great potential of 2D vdW heterostructures in enhancing and manipulating near-field thermal radiation, which may pave the way for highly efficient thermal management or designing and optimizing the energy conversion devices, such as thermophotovoltaics.

## 2. Theoretical Formalism

We assume that the 2D vdW heterostructures are suspended, i.e., free-standing without a supporting substrate, that the lateral dimensions are much larger than the characteristic thermal wavelength so that the finite size effect can be neglected, and that the thicknesses are negligible. Let us assume that they lie on the xy-plane, separated by a distance d, and each body is at thermal equilibrium held at temperatures $T_{1}$ and $T_{2}$ respectively. In the framework of fluctuational electrodynamics, combined with the dyadic Green’s functions, the near-field radiative heat flux $Q\left(T_{1},T_{2};d\right)$ between two sheets can be expressed in a Landauer-like formula [49]:

(1) $$\begin{array}{cc} Q\left(T_{1},T_{2};d\right) & |=\int_{0}^{\infty }\frac{d\omega }{2\pi }q\left(\omega \right)\\ & |=\int_{0}^{\infty }\frac{d\omega }{2\pi }\left[\Theta \left(\omega ,T_{2}\right)-\Theta \left(\omega ,T_{1}\right)\right]\int_{0}^{\infty }\frac{kdk}{{\left(2\pi \right)}^{2}}T\left(\omega ,k;d\right), \end{array}$$

Here, $q\left(\omega \right)$ denotes the spectral heat flux and $\Theta \left(\omega ,T\right)=\frac{\hbar \omega }{\left[\exp\left(\frac{\hbar \omega }{k_{B}T}\right)-1\right]}$ is the average energy of Plank oscillators. The integral over the wave vector k of the second term on the right-hand side of Equation (1) is defined as the spectral energy transfer function $\Phi \left(\omega \right)$. $T\left(\omega ,k;d\right)$ is the energy transmission coefficient, given by [27,50]:

(2) $$T\left(\omega ,k;d\right)=\left\{\begin{array}{c} |\mathrm{Tr}\left[\left(I-R_{2}^{†}R_{2}\right)D_{12}\left(I-R_{1}R_{1}^{†}\right)D_{12}^{†}\right]\\ |\mathrm{Tr}\left[\left(R_{2}^{†}-R_{2}\right)D_{12}\left(R_{1}-R_{1}^{†}\right)D_{12}^{†}\right]e^{-2\left|k_{z0}\right|d} \end{array}\right.\;\;\begin{array}{c} k<k_{0}\\ k>k_{0} \end{array}$$

where $k_{0}=\frac{\omega }{c}$ represents the vacuum wave vector; $k_{z0}=\sqrt{k_{0}^{2}-k^{2}}$ and $k=\sqrt{k_{x}^{2}+k_{y}^{2}}$ are the wave vector components perpendicular and parallel to the interface in a vacuum, respectively. $D_{12}={\left(I-R_{1}R_{2}e^{2ik_{z0}d}\right)}^{-1}$ denotes the Fabry-Perot-like denominator matrix due to multiple reflections. $R_{i}$ is the reflection coefficient matrix from vacuum to object i, written as

(3) $$R_{i}=\left[\begin{array}{cc} r_{i}^{\mathrm{ss}} & r_{i}^{\mathrm{sp}}\\ r_{i}^{\mathrm{ps}} & r_{i}^{\mathrm{pp}} \end{array}\right]$$

where $r_{i}^{mn}\left(i=1,2\right)$ is the total effective reflection coefficient from the objects i, m, and n indicate the polarization states (*p*- or *s*-polarization) of the incident and reflected electromagnetic waves. In this paper, only symmetric structures of cold and hot sides are considered, so $R_{1}=R_{2}$.

In this paper, all materials are non-magnetic, then $r_{1}^{\mathrm{sp}}=r_{1}^{\mathrm{ps}}=0$. For the suspended 2D sheets, the Fresnel reflection coefficients can be given by:

(4) $$r_{1}^{\mathrm{ss}}=\frac{-\omega \mu_{0}\sigma }{2k_{z0}+\omega \mu_{0}\sigma }\;r_{1}^{\mathrm{pp}}=\frac{\sigma }{\frac{2\omega \varepsilon_{0}}{k_{z0}}+\sigma }$$

where $k_{z0}={\left(k_{0}^{2}\varepsilon_{⊥}-k^{2}\right)}^{1/2}$ represent the z-component wavevector in a vacuum and σ is the optical conductivity of the 2D sheets. By simply substituting the optical conductivity of the 2D vdW heterostructure into the above equations, we can obtain the near-field thermal radiation characteristics.

### Optical Properties

The 2D vdW heterostructure in this study is considered as a random stack of monolayer graphene and 2D hBN, and neglecting thickness, its effective conductivity can be obtained by simply adding the conductivities of the two components [51]: $\sigma_{\mathrm{eff}}=\sigma_{G}+\sigma_{2D-\mathrm{hBN}}$, where $\sigma_{G}$ and $\sigma_{2D-\mathrm{hBN}}$ are optical conductivities of graphene and 2D hBN, respectively. In the local approximation, the optical conductivity of graphene can be written as $\sigma_{G}=\sigma_{D}+\sigma_{I}$, where $\sigma_{D}$ and $\sigma_{I}$ represent the contributions of intraband transitions (given by the Drude model) and interband transitions, respectively, written as [52]:

(5) $$\sigma_{D}=\frac{i}{\omega +\frac{i}{\tau }}\frac{e^{2}2k_{B}T}{\pi \hbar^{2}}\ln\left(2\cos h\frac{\mu }{2k_{B}T}\right),$$

(6) $$\sigma_{I}=\frac{e^{2}}{4\hbar }\left[G\left(\frac{\hbar \omega }{2}\right)+i\frac{4\hbar \omega }{\pi }\int_{0}^{\infty }\frac{G\left(x\right)-G\left(\frac{\hbar \omega }{2}\right)}{{\left(\hbar \omega \right)}^{2}-4x^{2}}dx\right]$$

Here, $G\left(x\right)=\frac{\sin h\left(\frac{x}{k_{B}T}\right)}{\left[\cos h\left(\frac{\mu }{k_{B}T}\right)+\cos h\left(\frac{x}{k_{B}T}\right)\right]}$. μ is the chemical potential. The optical conductivity of 2D hBN is given by [43]:

(7) $$\sigma_{2D-\mathrm{hBN}}=\frac{-4i\varepsilon_{0}\varepsilon_{\mathrm{env}}\omega \omega_{\mathrm{TO}}v_{g}}{\omega_{\mathrm{TO}}^{2}-\omega^{2}-i\omega \tau^{-1}}$$

where $\varepsilon_{0}$ is the dielectric function of free space, $\varepsilon_{\mathrm{env}}$ is the average dielectric function of the bulk material above and below the 2D hBN, $v_{g}$ is the LO phonon group velocity, and $\omega_{\mathrm{TO}}$ is the transverse optical phonon frequency. $\sigma_{2D-\mathrm{hBN}}$ includes the $\varepsilon_{\mathrm{env}}$ term, but actually not affected by it because $v_{g}$ is inversely proportional to $\varepsilon_{\mathrm{env}}$. Therefore, for simplicity, we set $\varepsilon_{\mathrm{env}}=1$ in this paper. Other parameters are taken from [42,43]: $\tau^{-1}=5\;{\mathrm{cm}}^{-1}$, and $v_{g}=1.2\times 10^{-4}c_{0}$, where $c_{0}$ is the light speed in a vacuum.

**Figure 1:**
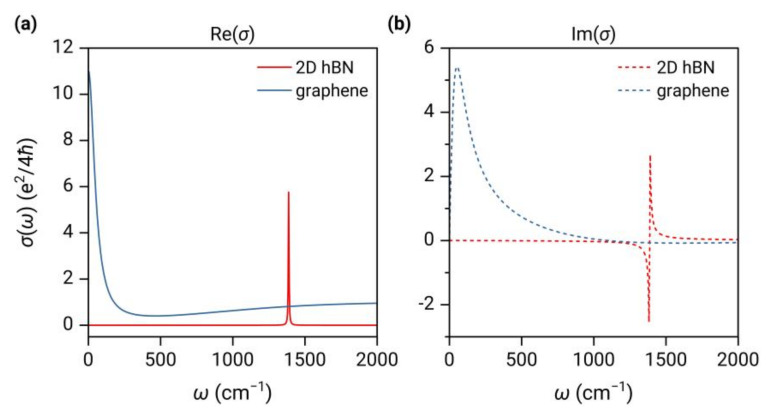
(**a**) The real parts and (**b**) the imaginary parts of the conductivity of graphene and 2D hBN as a function of frequency. The chemical potential of graphene is set to μ=0.05 eV and the temperature is 300 K.

The real and imaginary parts of the optical conductivity of graphene and 2D hBN as a function of frequency are plotted in Figure 1. It is shown that for graphene at μ=0.05 eV, the imaginary part ImσG shows a strong peak at very low frequencies, mainly dominated by the intraband transitions, and the real part gradually decreases and converges to the universal conductivity $\sigma_{0}=\frac{e^{2}}{\left(4\hbar \right)}$ for frequencies above the threshold of the interband transition (ℏω>2μ=0.1 eV, i.e., $\omega >807{\;\mathrm{cm}}^{-1}$). On the other hand, apart from the sharp peak at $\omega =\omega_{\mathrm{TO}}=1387\;{\mathrm{cm}}^{-1}$, 2D hBN exhibits a nearly constant value close to zero at other frequencies.

## 3. Results and Discussion

Based on the previous theoretical formalism, we performed numerical calculations of the near-field thermal radiation using MATLAB code.

### 3.1. Energy Transmission Coefficient and Dispersion Relation

**Figure 2:**
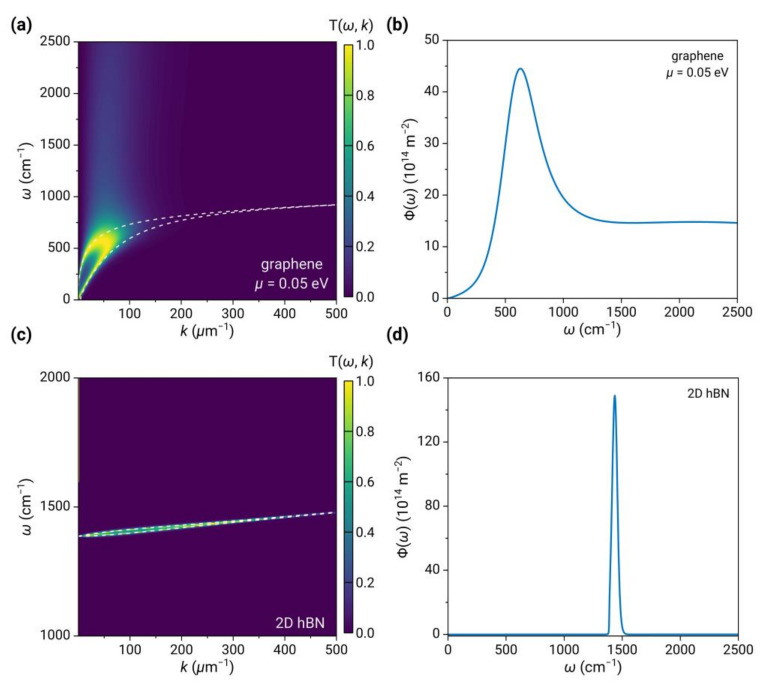
(**a**,**c**) The near-field radiative energy transmission coefficient $T\left(\omega ,k;d\right)$ and (**b**,**d**) energy transfer function $\Phi \left(\omega \right)$ for two graphene sheets (top panels) and two 2D hBN sheets (top panels). The temperatures are 290 K and 300 K, the distance is 10 nm, and the chemical potential of graphene is 0.05 eV. The white dashed curves are dispersion relations.

To understand the near-field thermal radiation of 2D vdW heterostructures, we first separately calculated the near-field radiative characteristics of graphene and 2D hBN. In Figure 2, we plot the energy transmission coefficient $T\left(\omega ,k;d\right)$ and energy transfer function $\Phi \left(\omega \right)$ for the case of two graphene sheets (top panels) and two 2D hBN sheets (top panels), respectively, with a vacuum gap of 10 nm and the chemical potential of μ=0.05 eV.

For the case of graphene, two bright bands shown in the low-frequency region ($\omega <1000{\;\mathrm{cm}}^{-1}$) of $T\left(\omega ,k;d\right)$ (see Figure 2a), starting from the origin and converging at $k\approx 100\;\mu m^{-1}$, correspond to the coupled plasmons between the two graphene sheets, including the high-frequency optical branch and the low-frequency acoustic branch. The dispersion relations of plasmons can be obtained by the pole of the reflection coefficient, given by:

(8) $$1+\frac{\sigma k_{z0}}{\omega \varepsilon_{0}}=\coth\left(\frac{ik_{z0}d}{2}\right)\;1+\frac{\sigma k_{z0}}{\omega \varepsilon_{0}}=\tanh\left(\frac{ik_{z0}d}{2}\right)$$

As shown by the dashed curves, the plasmon polaritons give rise to strong light absorption at the interface, thus resulting in a maximum value of $T\left(\omega ,k\right)=1$. In addition, there is also a less bright band at high frequencies due to the contribution of inter-band transitions of graphene. This band starts from the threshold frequency of inter-band transition (ℏω=2μ=0.1 eV, i.e., $\omega \approx 807{\;\mathrm{cm}}^{-1}$) and is almost independent of the wave vector. As a result, the spectrum of $\Phi \left(\omega \right)$ (see Figure 2b) shows a strong peak resulting from the contribution of intraband transitions (plasmons) and a virtually flat, broad band at high frequencies resulting from the contribution of interband transitions.

Compared to the broadband behavior of graphene, in the case of 2D hBN, as shown in Figure 2c,d, the contour of $T\left(\omega ,k\right)$ displays a very narrow bright band around $\omega_{\mathrm{TO}}=1387{\;\mathrm{cm}}^{-1}$, almost dispersionless, with its maximum wavevector contributing to the spectrum much larger than that of graphene plasmons. This results from the 2D phonon-polaritons (LO phonons) in 2D hBN, which dispersion can also be described by Equation (8) by replacing the conductivity with that of 2D hBN. Therefore, the $\Phi \left(\omega \right)$ spectrum exhibits an extremely narrow peak around $\omega_{\mathrm{TO}}=1387{\;\mathrm{cm}}^{-1}$, with a full width at half maximum of $\sim 50{\;\mathrm{cm}}^{-1}$, and a peak intensity nearly four times higher than that of graphene, while the rest of the spectrum is almost negligible.

**Figure 3:**
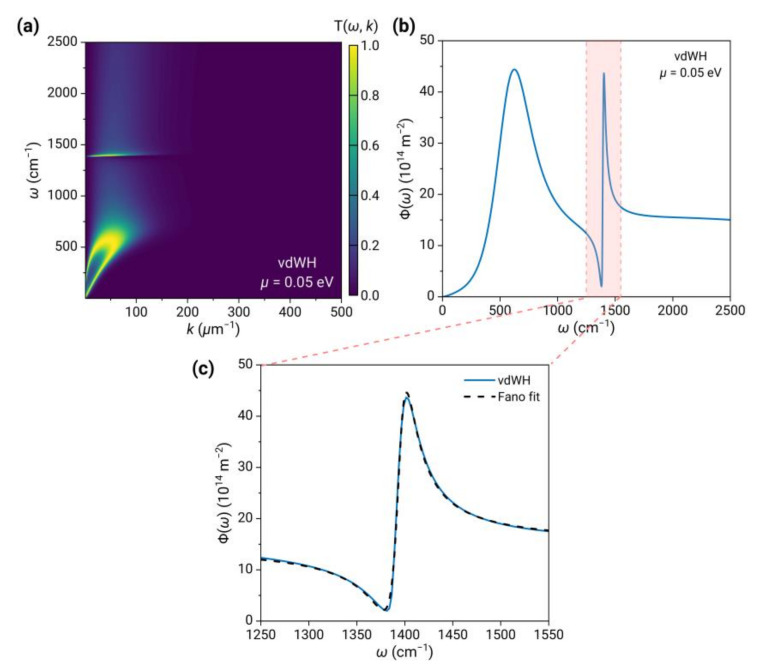
(**a**,**b**) The energy transmission coefficient $T\left(\omega ,k;d\right)$ and energy transfer function $\Phi \left(\omega \right)$ between two 2D vdW heterostructure sheets at temperatures of 290 K and 300 K. The distance is 10 nm, and the chemical potential of graphene is 0.05 eV. (**c**) The zoom-in view of $\Phi \left(\omega \right)$ on the shaded region in (**b**) and the fitting using the Fano formula.

When graphene and 2D hBN are randomly stacked to form a 2D vdW heterostructure, the phonon polaritons in 2D hBN and the plasmons in graphene are far apart and do not couple directly; therefore, the energy transmission coefficient $T\left(\omega ,k;d\right)$ is nearly equal to the direct sum of those of the two materials, except for a significantly reduced maximum wavevector of phonon polaritons, as shown in Figure 3a. Similarly, the spectrum $\Phi \left(\omega \right)$ (see Figure 3b) is found to closely resemble that of graphene, barring an asymmetric spectral peak in the vicinity of $\omega_{\mathrm{TO}}$ that is typical of Fano interference. Notably, at this frequency, phonon-polariton resonance generates a narrowband spectral peak in 2D hBN, whereas graphene exhibits a broadband and nearly flat spectrum. The emergence of the asymmetrical spectral peak is attributed to the interaction between the two states. To verify the Fano resonance, we fit the spectrum near $\omega_{\mathrm{TO}}$ using the Fano formula [46]:

(9) $$\sigma \left(E\right)=D^{2}\frac{{\left(q+\Omega \right)}^{2}}{1+\Omega^{2}}$$

where E is the energy, q=cotδ is the Fano parameter, δ is the phase shift of the continuum; $\Omega =\frac{2\left(E-E_{0}\right)}{\Gamma }$, where $E_{0}$ and Γ are the resonance energy and width, respectively; $D^{2}=4\sin^{2}\delta$. As shown in Figure 3c, there is good agreement between the fitting and calculation results, thereby corroborating the Fano interference responsible for the emergence of the asymmetrical spectral line shape. The fundamental physical mechanism can be elucidated by the spectral interference between a narrowband discrete resonance (i.e., the 2D hBN phonon polariton) and a broadband continuum state (i.e., the contribution of interband transitions in graphene).

### 3.2. Effect of Chemical Potential of Graphene

**Figure 4:**
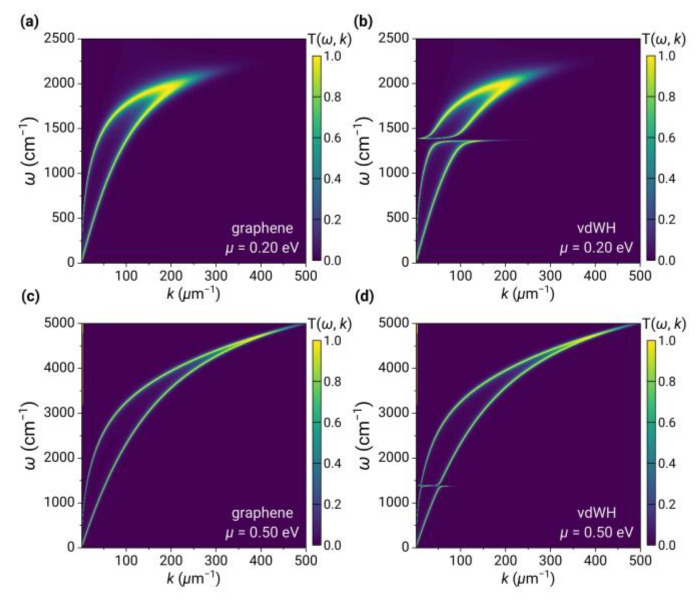
Energy transmission coefficient $T\left(\omega ,k;d\right)$ for graphene (left panels) and 2D vdW heterostructures (right panels) at chemical potentials of (**a**,**b**) 0.2 eV and (**c**,**d**) 0.5 eV.

The optical property and plasmonic behavior of graphene can be modulated by changing the chemical potential through chemical doping or electrical gating. In Figure 4, we plot the contour of $T\left(\omega ,k;d\right)$ for graphene and 2D vdW heterostructures at different chemical potentials of 0.2 eV and 0.5 eV, respectively, as a function of wave vector and frequency.

As the chemical potential of graphene increases (compared to μ=0.05 eV in Figure 2), the interband transitions threshold (ℏω=2μ) exceeds the frequency range of our interest. Therefore, the plasmons of intraband transitions dominate, resulting in increased bandwidth and maximum wavevector. In the case of 2D vdW heterostructure at μ=0.2 eV, a clear anti-crossing behavior in the bright band of $T\left(\omega ,k;d\right)$ was observed, as shown in Figure 4b, which can be attributed to the stronger coupling between the plasmons of graphene and the phonon polariton of 2D hBN. When the chemical potential increased to 0.5 eV, a weak anti-crossing behavior was observed, as shown in Figure 4d.

**Figure 5:**
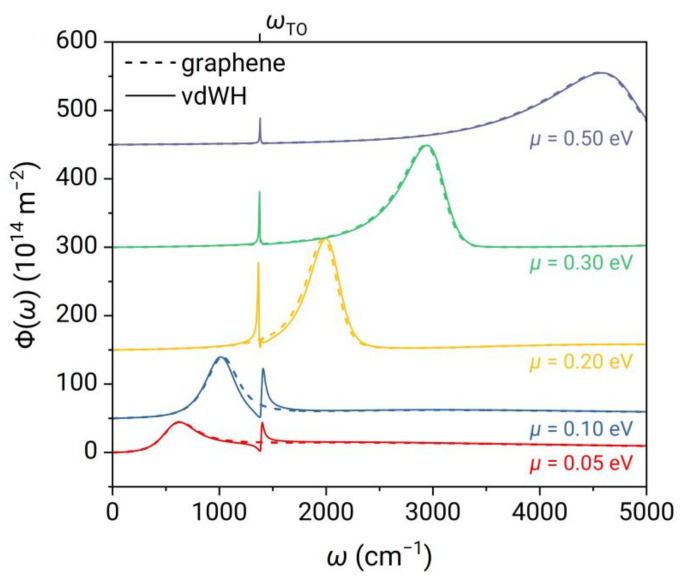
Energy transfer function $\Phi \left(\omega \right)$ as a function of frequency for 2D vdW heterostructure and monolayer graphene at different chemical potentials.

In Figure 5, we plot the spectra of $\Phi \left(\omega \right)$ for 2D vdW heterostructure and monolayer graphene at different chemical potentials. It is shown that graphene exhibits a single broadband peak due to surface plasmons. As the chemical potential increases, the peak frequency undergoes a blue shift, and the peak width gradually increases while the peak intensity initially increases and then decreases. In the case of the 2D vdW heterostructure at various chemical potentials, the overall spectra are almost identical to that of graphene, except for the narrow peak with asymmetric line shape at the frequency of $\omega_{\mathrm{TO}}$. As the chemical potential increases to $\mu >\frac{\hbar \omega_{\mathrm{TO}}}{2}\approx 0.086\;\mathrm{eV}$, the intraband Drude plasmon in graphene begins to couple with the 2D phonon polaritons in 2D hBN, and the two resonance peaks gradually approach each other. When the chemical potential is further increased, the resonance frequency of the former exceeds that of the latter, and the two peaks gradually move apart, weakening the coupling.

**Figure 6:**
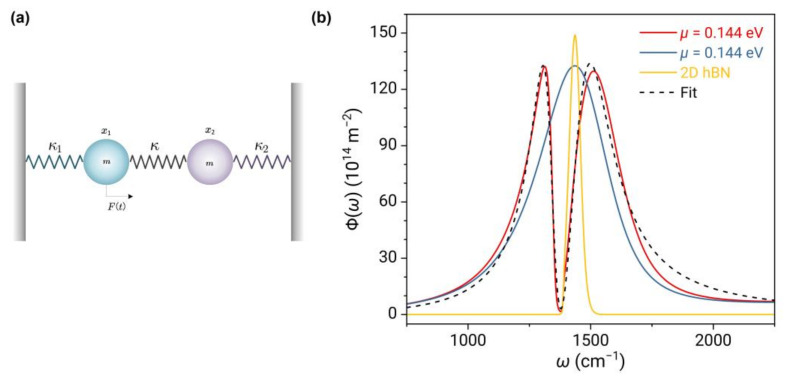
(**a**) Coupled oscillator model. $\kappa_{1}$, $\kappa_{2}$, and κ are spring constants, m is the mass and $F\left(t\right)$ is the driving force. (**b**) Spectra of energy transfer function $\Phi \left(\omega \right)$ of graphene (blue curve), 2D hBN (yellow curve), and 2D vdW heterostructure (red curve) at μ=0.144 eV. The dashed curve represents the fitted curve using Equation (11).

The absorption spectrum resulting from the coupling between the broadband plasmon resonance in graphene and the narrowband phonon-polariton resonance in 2D hBN can be qualitatively described using a classical coupled oscillator model [53,54], as shown in Figure 6a. The expression for this model is given below:

(10) $$\begin{array}{c} {\ddot{x}}_{1}+\gamma_{1}{\dot{x}}_{1}+\omega_{1}^{2}x_{1}-\Omega^{2}x_{2}=\frac{F}{m}\exp\left(-i\omega_{s}t\right)\\ {\ddot{x}}_{2}+\gamma_{2}{\dot{x}}_{2}+\omega_{2}^{2}x_{2}-\Omega^{2}x_{1}=0 \end{array}$$

Here, $x_{1}$ and $x_{2}$ are displacements with respect to their equilibrium positions; $\omega_{1}$ and $\omega_{2}$ are the frequency of the two oscillators without coupling; $\Omega^{2}=\frac{K}{m}$ is the frequency associated with the coherent coupling between two oscillators with K is the coupling spring constant; F is the driving force. The absorption spectrum of oscillator 1 can be obtained as [53,55]:

(11) Psωs=Re−2πiF2ωsω22−ωs2−iγ2ωsω12−ωs2−iγ1ωsω22−ωs2−iγ2ωs−Ω4

when the two oscillators have different damping rates, and the coupling strength is smaller than the larger damping rate, an asymmetric and sharp Fano resonance appears, causing a dramatic change between the peak and the valley in the absorption spectrum, as shown near the frequency $\omega_{\mathrm{TO}}$ in Figure 5.

When the detuning frequency is 0, i.e., $\omega_{1}=\omega_{2}$, the lowest absorption is displayed at the resonant frequency (“transparency window”). This is known as electromagnetic-induced transparency, and it can be seen as a special case of Fano resonance. As shown in Figure 6b, when the chemical potential of graphene is 0.144 eV, the resonant frequencies of the two materials match, and their coupling is strongest. Two nearly symmetric spectral peaks emerge on either side of the resonant frequency, while a near-zero minimum value occurs at the resonant frequency, which is a typical feature of electromagnetically induced transparency. The curve fitted by Equation (11) agrees well with the calculated curve near the resonant frequency.

### 3.3. Spectra and Heat Flux

**Figure 7:**
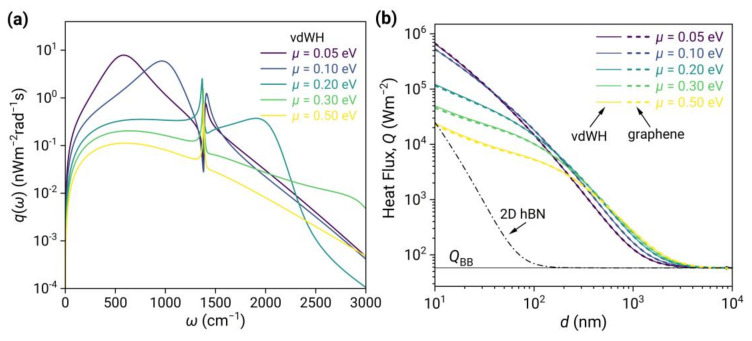
(**a**) Near-field radiative spectral heat flux $q\left(\omega \right)$ of 2D vdW heterostructures at different chemical potentials. (**b**) heat flux $Q\left(T_{1},T_{2};d\right)$ as a function of separation for 2D vdW heterostructures (solid curves) and graphene (dashed curves) at different chemical potentials, as well as 2D hBN (dash-dotted curves). The temperatures are 290 K and 300 K, respectively.

The preceding section primarily addressed the spectra of energy transfer functions $\Phi \left(\omega \right)$. In this section, we will examine the near-field radiative spectral heat flux $q\left(\omega \right)=\left[\Theta \left(\omega ,T_{2}\right)-\Theta \left(\omega ,T_{1}\right)\right]\Phi \left(\omega \right)$. The resonators’ energy distribution $\;\Theta \left(\omega ,T\right)$ follows an exponential decay with frequency, which serves as a low-pass filter in the spectrum and thereby enhances the weight of peaks at lower frequencies. Furthermore, the low-pass range expands with the rise in temperature. As shown in Figure 7a, at low chemical potentials, the plasmons in graphene result in a broad and strong peak in $q\left(\omega \right)$ that dominates the spectrum since it located at lower frequencies. In contrast, the peak in $\Phi \left(\omega \right)$ generated by phonon polaritons in 2D hBN is comparable to that of graphene but is one order of magnitude lower in $q\left(\omega \right)$. As the chemical potential increases, the plasmons shift to higher frequencies, and their weight significantly decreases. Although they are enhanced in $\Phi \left(\omega \right)$, they cannot compensate for the exponential decay of $\Theta \left(\omega ,T\right)$. Conversely, the contribution of phonon-polaritons in 2D hBN becomes more prominent, leading to only a narrowband peak in $q\left(\omega \right)$ for μ>0.2 eV.

In Figure 7b, we present a comparison of the near-field radiative heat flux for 2D vdW heterostructures (solid curves) and graphene (dashed curves) at different chemical potentials, as well as 2D hBN (dash-dotted curves). It is shown that 2D vdW heterostructures and graphene have nearly the same radiative heat flux, which can exceed the blackbody limit by up to five orders of magnitude in the near field. Additionally, this flux decreases significantly as the chemical potential of graphene increases at small distances because the spectral peak undergoes a blue shift, resulting in a decrease in weight. In comparison, the radiative heat flux of 2D hBN is significantly lower than both and decreases more rapidly with distance because the spectral peak of 2D hBN is narrow banded and at a higher frequency, resulting in a lower weight in $q\left(\omega \right)$.

To conclude, 2D vdW heterostructures can achieve the same high radiative heat flux as graphene, yet with a markedly different spectral distribution, particularly at high chemical potentials.

### 3.4. Effect of the Substrate

The previous considerations are mainly for suspended 2D vdW heterostructures; although it can be synthesized experimentally [56], it is more practical to have substrates in the experimental implementation to ensure the stability of the structure. Further, different substrates can bring different optical responses and, as well as physical mechanisms interacting with 2D vdW heterostructures. In this section, we discuss the effect of substrates.

**Figure 8:**
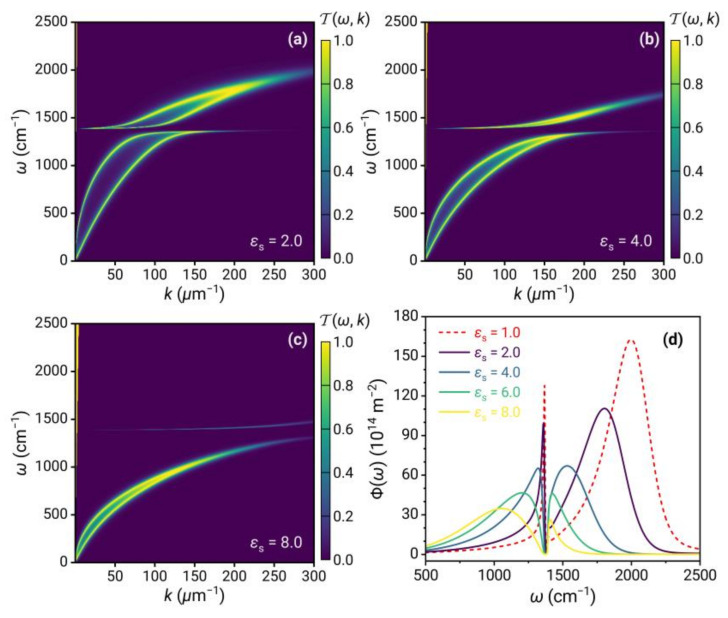
(**a**–**c**) Energy transmission coefficient $T\left(\omega ,k;d\right)$ and (**d**) energy transfer function $\Phi \left(\omega \right)$ for 2D vdW heterostructures covered on the substrate with different $\varepsilon_{s}$.

For simplicity, we first consider non-dissipative substrates with constant dielectric constants $\varepsilon_{s}$. The results of the energy transmission coefficient and energy transfer function are shown in Figure 8. The presence of the substrate also significantly affects the near-field thermal radiation. As the dielectric constant of the substrate increases, the frequency range of the graphene plasmons decreases significantly so that the high-frequency part, which is coupled with the phonon polaritons of 2D hBN, gradually disappears in the contour plot of the energy transmission coefficient. Therefore, the spectra of energy transfer function $\Phi \left(\omega \right)$ show that the broadband peak resulting from the plasmons is gradually redshifted, and the peak value is considerably decreased. During the redshift process, the plasmons are coupled with the narrowband resonance of the 2D hBN phonon polaritons, resulting in the spectrum transition from the Fano-type line shape to the electromagnetically induced transparency and then to the Fano line shape. Increasing the dielectric constant of the substrate has a similar effect as lowering the chemical potential of graphene.

**Figure 9:**
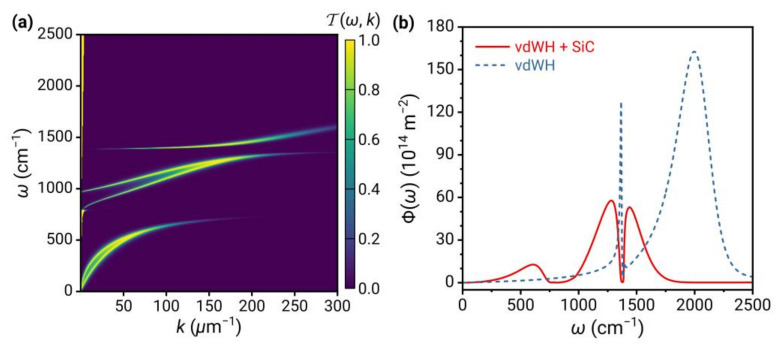
(**a**) The energy transmission coefficient $T\left(\omega ,k;d\right)$ and (**b**) energy transfer function $\Phi \left(\omega \right)$ for 2D vdW heterostructures covered on SiC substrate. The dashed curve is the result of suspended 2D vdW heterostructures.

For practical dissipative substrates, each substrate requires separate analysis because the optical response and the physical mechanism that interacts with the 2D vdW heterostructure are different for various materials (e.g., surface phonon polaritons in polar materials, surface plasmon polaritons in metals, etc.). As an example, we use the widely used SiC as a substrate for illustration, as shown in Figure 9. The optical property of SiC is given in Ref. [20].

The plasmons in graphene are coupled to the surface phonon polaritons of SiC and the 2D phonon of 2D hBN. Hence, in the contour plot of $T\left(\omega ,k;d\right)$, the bright bands are divided into three parts, with two clear anti-crossing behaviors caused by mode repulsion at the frequencies $\omega_{\mathrm{TO}}$ of SiC and 2D hBN. In the spectrum of $\Phi \left(\omega \right)$*,* three parts of the bright band give rise to three peaks, with a valley at the frequency $\omega_{\mathrm{TO}}=1387{\;\mathrm{cm}}^{-1}$ of 2D hBN and two nearly symmetrical peaks on either side, similar to the EIT phenomenon in Figure 6. In addition, the presence of the substrate leads to a substantial decrease in peak values compared to the suspended case, similar to Figure 8d.

### 3.5. Discussion

In this paper, we have shown that near-field thermal radiation of 2D vdW heterostructure can be significantly enhanced and modulated by tuning the chemical potential of graphene (e.g., it can be achieved experimentally by applying the gate voltage [35,36]). In addition to chemical potential, there are other factors that may affect the near-field thermal radiation spectrum. For example, the heterostructure discussed in this paper is randomly stacked (i.e., graphene and 2D hBN are considered as two separate 2D sheets), and its stacking forms (e.g., AA or AB) and the defects in realistic models may have an impact on its optical response. Furthermore, the mechanical strain may also be used to tune the thermal radiation of heterostructure, such as in hBN [57] and graphene [58]. In addition, finite size effects, as well as lateral boundaries, may also have an important impact on the near-field thermal radiation properties, which is an interesting topic worthy of further investigation. Although hBN is used as an example in this study, similar phenomena may be extended to other 2D polar materials [42] as well as to 2D perovskite oxides, such as 2D SrTiO_3_ and LiNbO_3_ [59], which have similar phonon polariton properties as 2D hBN.

## 4. Conclusions

In this paper, we investigate the near-field thermal radiation of 2D vdW heterostructures vertically stacked of graphene and monolayer polar material (2D hBN as an example). We show an asymmetric Fano resonance in the radiation spectrum resulting from the interference between a broad continuum of surface plasmon polaritons (SPPs) in graphene and a narrow discrete state of 2D phonon polaritons in 2D hBN. In addition, we show that 2D van der Waals heterostructures can achieve nearly the same high radiative heat flux as graphene but with markedly different spectral distributions, especially at high chemical potentials. By tuning the chemical potential of graphene, we can actively control the radiative heat flux of 2D van der Waals heterostructures and manipulate the radiative spectrum, such as the transition from Fano resonance to electromagnetic-induced transparency (EIT). Our results highlight the great potential of 2D vdW heterostructures to enhance and manipulate near-field thermal radiation, which may pave the way for highly efficient thermal management or design and optimization of energy conversion devices, such as thermophotovoltaics.

## Data Availability

The data presented in this study are available upon request from the corresponding author.
